# Colored Point Cloud Registration by Depth Filtering

**DOI:** 10.3390/s21217023

**Published:** 2021-10-23

**Authors:** Ouk Choi, Wonjun Hwang

**Affiliations:** 1 Department of Electronics Engineering, Incheon National University, Yeonsu-gu, Incheon 22012, Korea; ouk.choi@inu.ac.kr; 2 Department of Software and Computer Engineering and Department of Artificial Intelligence, Ajou University, Yeongtong-gu, Suwon 16499, Korea

**Keywords:** point cloud registration, ICP, depth filtering

## Abstract

In the last stage of colored point cloud registration, depth measurement errors hinder the achievement of accurate and visually plausible alignments. Recently, an algorithm has been proposed to extend the Iterative Closest Point (ICP) algorithm to refine the measured depth values instead of the pose between point clouds. However, the algorithm suffers from numerical instability, so a postprocessing step is needed to restrict erroneous output depth values. In this paper, we present a new algorithm with improved numerical stability. Unlike the previous algorithm heavily relying on point-to-plane distances, our algorithm constructs a cost function based on an adaptive combination of two different projected distances to prevent numerical instability. We address the problem of registering a source point cloud to the union of the source and reference point clouds. This extension allows all source points to be processed in a unified filtering framework, irrespective of the existence of their corresponding points in the reference point cloud. The extension also improves the numerical stability of using the point-to-plane distances. The experiments show that the proposed algorithm improves the registration accuracy and provides high-quality alignments of colored point clouds.

## 1. Introduction

RGB-depth (RGB-D) cameras are widely used for 3D modeling [1,2,3,4] and human pose estimation [5] due to their ability to acquire depth images in real time. RGB-D cameras provide color images aligned to the depth images, so each pixel location in a color–depth image pair is recorded with the 3D coordinates of a point and its RGB data. The 6D data enable the modeling of texture as well as structure.

To reconstruct the entire 3D structure of an object, one can use a single RGB-D camera to acquire an RGB-D video, moving the camera around the object [1,2]. If the object is dynamic, one can use a synchronized multiview RGB-D camera system [3,4]. In both cases, estimating the 3D rigid transformation across point clouds is the key problem to solve to obtain a single merged point cloud. If the frame rate of the RGB-D video is high, the identity transformation can be regarded as the initial estimate [1]. For the multiview system, either extrinsic calibration [6,7] or global registration algorithms [8,9,10,11] can be employed.

The remaining errors in the transformations are effectively reduced by the Iterative Closest Point (ICP) algorithm [12,13,14] and its variants [15,16,17,18,19,20,21,22,23,24,25,26]. The ICP algorithm registers a source point cloud to a reference point cloud by repeatedly alternating steps of correspondence search and cost minimization. The correspondence search step transforms all source points to the reference frame using the current pose and then finds from the reference point cloud the closest point to each transformed source point. The point pairs whose point-to-point distance is shorter than a threshold are regarded as correspondences. The cost minimization step estimates the refined pose by minimizing a cost constructed from the correspondences.

The ICP variants [15,16,17,18,19,20,21,22,23,24,25,26] have improved the original algorithm by solving different problems, such as disambiguation of the correspondence search [15,21,24,26], defining a better cost function [13,16,17,18,19,20,22,23,25,26], and searching for a better optimization method [16,17,18,19,25]. Even with accurate poses, the registration accuracy is limited by the random and systematic depth measurement errors of the RGB-D cameras [27]. The depth errors also lead to the poor visual quality of the merged point cloud. Reducing the errors in the earliest stage of the pipeline can wipe out the local structure, which is essential for the correspondence search. For this reason, depth-error reduction is often the last stage of the pipeline [8,26].

Simple postprocessing on the merged point cloud filters each 3D point using its neighbors [28]. If the poses are inaccurate, only the neighbors from the same fragment tend to have large weights. In this case, the accuracy of individual point clouds can be improved; however, corresponding points across point clouds may not mix to produce a seamlessly merged point cloud. On the other hand, the cost functions of the ICP algorithm and its variants are designed to minimize the distance between corresponding points across point clouds. Thus, the registration can become more accurate by minimizing the cost further. A recent study showed that the cost of an ICP algorithm can be minimized further by refining the measured depth values instead of the pose parameters [26]. However, the depth-update equation derived from the cost function tends to be numerically unstable, so a postprocessing step is needed to restrict the range of the output depth values. In addition, the points outside of the overlapping surfaces between point clouds are not covered by the cost function, so the depth errors of those points are not reduced by minimizing the cost function. As a solution, a regularization method is applied at the final step.

In this paper, we present a new cost function that is not only stable to minimize but also applied to all source points, irrespective of their corresponding points in the reference point cloud. We provide the reasoning for the unstable case of using the point-to-plane distance [26], where a 3D point-to-point vector is projected onto the surface-normal direction. To prevent the unstable case, our cost function is built on an adaptive combination of two different projected distances instead of a single projected distance.

Another contribution of this paper is that we consider the problem of registering a source point cloud to the union of the source and reference point clouds. The source points without their closest points in the reference point cloud will have their closest points within the source point cloud as long as the distance threshold permits. This extension allows all points to be processed in a unified filtering framework. Unlike the filtering approach in [28], the closest points are independently collected from the source and reference point clouds, and the effect of each set of closest points is controlled with a single parameter in our approach. Thus, we can control the mixing across point clouds.

The experimental results in this paper show that our proposed method prevents the unstable case, reduces the registration error, and provides high-quality merged point clouds. The results also show that the intra-point-cloud closest points are effective not only for reducing the depth errors but also for improving numerical stability.

The remainder of this paper is structured as follows. The following section provides a summary of existing methods. Our proposed method is presented in Section 3. The experimental results are provided in Section 4. Finally, Section 5 concludes the paper.

## 2. Related Work

Kinect sensors are among the most widely used RGB-D cameras, which rely on either the structured light-pattern projection or the Time-of-Flight technology [29]. Irrespective of the technology, the standard deviation of the random depth errors increases with the depth of the subject. For the structured light-pattern projection technology, the standard deviation approximately increases with the squared depth of the subject [29]. For the Time-of-Flight technology, the standard deviation increases with the inverse of the amplitude of the received infrared light signal [30]. The RGB-D cameras used in our work are based on the structured light-pattern projection as in Kinect v1 sensors, sharing similar depth-error characteristics.

For the global registration of point clouds, geometric invariants are used to establish pose hypotheses [8,9], or histogram features [31] are used to establish candidate matches [11]. The global registration algorithms typically find solutions by minimizing cost functions, for which robust, fast, and accurate optimization is crucial. The RANSAC algorithm [32] is used in [8], and smart indexing data organization is used for the acceleration [9] of the optimization [8]. In [11], the graduated nonconvexity algorithm is applied only to the candidate matches for fast and accurate global registration of the point clouds.

The original ICP algorithm [12] has room for improvement, and many local registration algorithms [13,14,15,16,17,18,19,20,21,22,23,24,25,26] have been proposed by addressing different problems of the original algorithm. Setting the threshold appropriately in the correspondence-search step is important to collect sufficient correspondences while rejecting outliers. The threshold can be determined by using data statistics [14]. Alternatively, the effect of the outliers can be weakened by using a robust loss function [18] or a cost function based on sparsity-inducing norms [23].

If the initial pose is inaccurate, the correspondence-search step based only on the 3D distance is prone to error. To improve the correspondence search, the color distance between points can be used as an auxiliary measure, extending the 3D search to a 4D or 6D search [15,21,24,26].

If the density of the point clouds is low or the initial pose is inaccurate, finding one-to-one correspondence is neither exact nor accurate. From this point of view, probabilistic approaches [16,17,19] allow a source point to match all points in the reference point cloud, assigning matching probabilities to all the correspondences. The annealing schedule of the matching probability distribution allows all the correspondences to be equally probable at the beginning of the iterations and preserves only dominant one-to-one correspondences at the end of the iterations [16,17]. To reduce the computational complexity of the probabilistic approaches, a coarse-to-fine scheme [25] can be used or the probabilities can be assigned only to the *K*-closest points [26], which can be efficiently obtained using a *K*D tree [33].

The original ICP algorithm relies on a cost function, which is the sum of squared point-to-point distances [12]. Chen and Medioni proposed to use a different cost function based on point-to-plane distances [13]. To compute the point-to-plane distance between a source point and a reference point, the difference vector between the points is projected onto the surface-normal vector of the reference point. The projected distance is equivalent to a Mahalanobis distance induced by a 3×3 matrix, which is the outer product of the surface-normal vector. Segal et al. [20] show that point-to-plane and plane-to-plane distances can be represented by Mahalanobis distances. The Mahalanobis distance can also be used to reflect the anisotropic, inhomogeneous localization error of the measured points [22]. Park et al. [25] use a cost function based on both color and depth differences between two point clouds.

Deformable ICP algorithms change the individual point locations as well as the pose of the source point cloud [34,35,36]. The algorithms assume that the object is deformable or articulated. In contrast, we assume that the multiview system is synchronous, so the object is assumed to be rigid across point clouds.

Our proposed method can be regarded as the unification of depth-error reduction [30,37] and point cloud registration [26]. Depth-error reduction algorithms refine measured depth values using the neighborhood within a depth image [30,37]. The Iterative *K* Closet Point (IKCP) algorithm [26] refines measured depth values using the *K*-closest points across point clouds. Our proposed method exploits the advantage of using the closest points from both source and reference point clouds.

Our method is similar to the bilateral filter for point clouds [28] in that it changes the 3D position of a point using its neighbors. However, our method has several differences from the bilateral filter. One difference is the direction of change of the 3D point. Each point moves along the surface-normal direction in the bilateral filter, whereas in our method, it moves along the ray direction so that the changed 3D point position matches the original pixel location in the depth image. Another difference is that our method uses color information, unlike the bilateral filter.

## 3. Proposed Method

In this section, we first review the Iterative *K* Closest Point (IKCP) algorithm [26] and then present our proposed method addressing the problems of the IKCP algorithm.

Let us denote the source and reference point cloud by $S_{s}$ and $S_{r}$, respectively, where $S_{s}={\left\{X_{i}^{\left(s\right)}\right\}}_{i=1}^{N_{s}}$ and $S_{r}={\left\{X_{i}^{\left(r\right)}\right\}}_{i=1}^{N_{r}}$. We assume that the 3D rigid transformation from a source point $X_{i}^{\left(s\right)}$ to its corresponding reference point $X_{j}^{\left(r\right)}$ has been given by the registration pipeline. The transformation is represented by a 3×3 rotation matrix R and a translation vector T:

(1) $$X_{j}^{\left(r\right)}=RX_{i}^{\left(s\right)}+T.$$

Defining ${\hat{X}}_{i}^{\left(s\right)}$ as ${\hat{X}}_{i}^{\left(s\right)}=RX_{i}^{\left(s\right)}+T$, a residual vector $d_{i,j}$ can be computed as $d_{i,j}=X_{j}^{\left(r\right)}-{\hat{X}}_{i}^{\left(s\right)}$. The IKCP algorithm for depth refinement aims at minimizing the following cost function.

(2) $$E=\sum_{i=1}^{N_{s}}E_{i},$$

where

(3) $$E_{i}=\sum_{j\in N_{i}}p_{i,j}d_{i,j}^{T}M_{i,j}d_{i,j}.$$

In Equation (3), $N_{i}$ is the index set of the *K*-closest points to ${\hat{X}}_{i}^{\left(s\right)}$. The *K*-closest points are searched for from $S_{r}$ with a constraint that requires $∥d_{i,j}∥$ to be less than a threshold τ. Thus, the cardinality of $N_{i}$ can be less than *K* according to the magnitude of $∥d_{i,j}∥$ and the setting of τ. $p_{i,j}$ is the weight of the correspondence between ${\hat{X}}_{i}^{\left(s\right)}$ and $X_{j}^{\left(r\right)}$, which is defined to decrease with the color-depth 6D difference between the two points. Finally, $M_{i,j}$ is a 3×3 matrix determined by the type of the distance. For example, $M_{i,j}=n_{j}n_{j}^{T}$ if the distance type is point-to-plane, where $n_{j}$ is the surface-normal vector of $X_{j}^{\left(r\right)}$. For the point-to-point distance, $M_{i,j}$ is simply the 3×3 identity matrix.

By regarding the depth $Z_{i}^{\left(s\right)}$ of $X_{i}^{\left(s\right)}={\left(X_{i}^{\left(s\right)},Y_{i}^{\left(s\right)},Z_{i}^{\left(s\right)}\right)}^{T}$ as a variable and R and T as fixed variables, Choi et al. [26] derived the following updated equation for minimizing *E*:

(4) $$Z_{i}^{\left(s\right)}\leftarrow \frac{x_{i}^{T}R^{T}\sum_{j\in N_{i}}p_{i,j}M_{i,j}\left(X_{j}^{\left(r\right)}-T\right)}{x_{i}^{T}R^{T}\sum_{j\in N_{i}}p_{i,j}M_{i,j}Rx_{i}},$$

where $x_{i}$ is the normalized image coordinates of $X_{i}^{\left(s\right)}$ satisfying $X_{i}^{\left(s\right)}=Z_{i}^{\left(s\right)}x_{i}^{\left(s\right)}$.

Denoting $\sum_{j\in N_{i}}p_{i,j}M_{i,j}$ by $M_{i}$, the update equation can become numerically unstable if $Rx_{i}$ is nearly in the null space of $M_{i}$. In [26], to improve the numerical stability, $M_{i,j}$ is defined as $M_{i,j}=\epsilon I+n_{j}n_{j}^{T}$, where ϵ is a small positive number. However, adding ϵI to $M_{i,j}$ does not completely prevent unwanted large changes in depth values, so Choi et al. [26] rely on a postprocessing step that restricts large changes.

**Figure 1:**
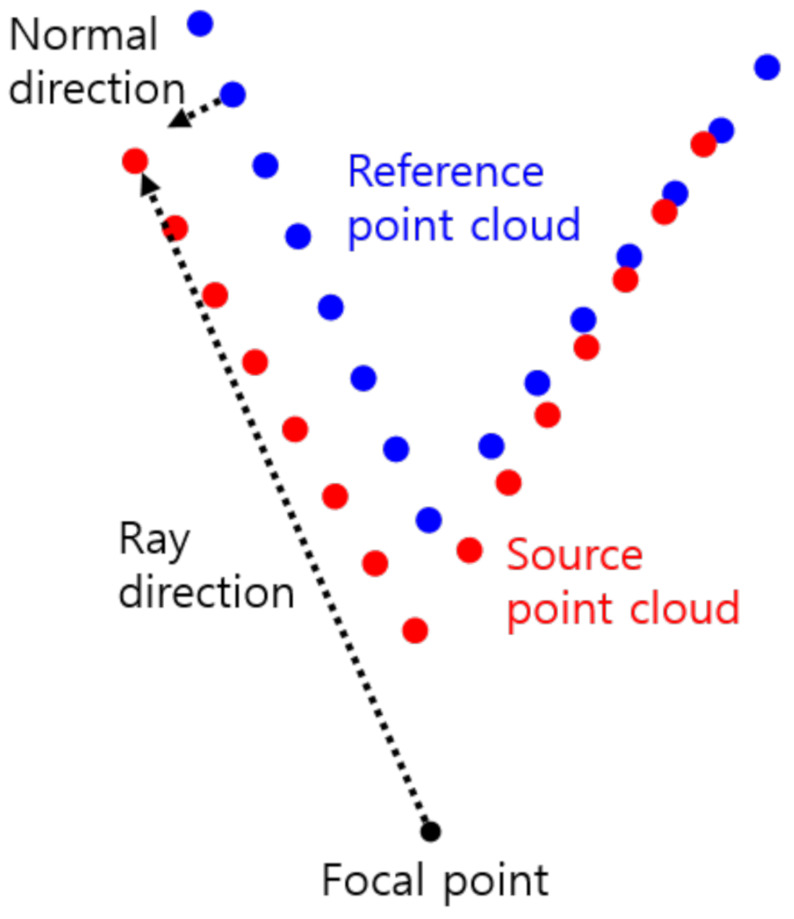
An unstable case in which the point-to-plane distance is hard to minimize by moving the source point in the ray direction. The red and the blue points represent a source and a reference point cloud, respectively. The ray direction is nearly orthogonal to the surface-normal direction.

In the IKCP algorithm, such a numerically unstable case occurs when the ray direction of a source point is nearly orthogonal to the dominant surface-normal direction of the *K*-closest points in the reference point cloud, as illustrated in Figure 1. As the source point is allowed to move only in the ray direction, the point-to-plane distance is difficult to decrease in such a case.

Let us assume that $p_{i,j}$ is very large for a certain reference point. Denoting the index of the point by $j^{⋆}$, the dominant surface-normal direction is $n_{j^{⋆}}$, and the matrix $M_{i}$ is approximately $\epsilon I+n_{j^{⋆}}n_{j^{⋆}}^{T}$. Assuming that the ray direction $Rx_{i}$ is nearly orthogonal to $n_{j^{⋆}}$, Equation (4) is approximately

(5) $$Z_{i}^{\left(s\right)}\leftarrow \frac{\epsilon x_{i}^{T}R^{T}\left(X_{j^{⋆}}^{\left(r\right)}-T\right)+x_{i}^{T}R^{T}n_{j^{⋆}}n_{j^{⋆}}^{T}\left(X_{j^{⋆}}^{\left(r\right)}-T\right)}{\epsilon ∥Rx_{i}{∥}^{2}}.$$

According to our assumption, the absolute value of $x_{i}^{T}R^{T}n_{j^{⋆}}$ is very small; however, $n_{j^{⋆}}^{T}\left(X_{j^{⋆}}^{\left(r\right)}-T\right)$ may not be negligible. Thus, with a small value of ϵ, the absolute value of $x_{i}^{T}R^{T}n_{j^{⋆}}n_{j^{⋆}}^{T}\left(X_{j^{⋆}}^{\left(r\right)}-T\right)$ may become non-negligible compared to the denominator, causing the computation of Equation (4) to be numerically unstable.

An easy method for increasing the numerical stability is simply to use the point-to-point distance. In this case, Equation (4) is simplified to

(6) $$Z_{i}^{\left(s\right)}\leftarrow \frac{x_{i}^{T}R^{T}\sum_{j\in N_{i}}p_{i,j}\left(X_{j}^{\left(r\right)}-T\right)}{∥Rx_{i}{∥}^{2}},$$

where ϵ has been removed.

We propose an adaptive method that exploits the fact that the direction $r_{i}$, whose dot product with $Rx_{i}$ is never zero, is $Rx_{i}$ itself or its non-zero multiple. For our new definition of $M_{i,j}$, let us define $r_{i}$ as

(7) $$r_{i}=\frac{Rx_{i}}{∥Rx_{i}∥}.$$

We define $M_{i,j}$ as a linear combination of $r_{i}r_{i}^{T}$ and $n_{j}n_{j}^{T}$:

(8) $$M_{i,j}=\left(1-c_{i,j}\right)r_{i}r_{i}^{T}+c_{i,j}n_{j}n_{j}^{T},$$

where $c_{i,j}$ is the coefficient of $n_{j}n_{j}^{T}$.

To avoid the numerical instability, $c_{i,j}$ needs to be small if $n_{j}$ is nearly orthogonal to $r_{i}$. To fulfill this requirement, we define $c_{i,j}$ as

(9) $$c_{i,j}={\left(n_{j}^{T}r_{i}\right)}^{2},$$

where $n_{j}^{T}r_{i}$ is the cosine of the angle θ between $n_{j}$ and $r_{i}$. Thus, $c_{i,j}$ is $\cos^{2}\theta$, and $\sin^{2}\theta$ is $1-\cos^{2}\theta$ or $1-c_{i,j}$.

With our new definition of $M_{i,j}$, if $n_{j}$ is nearly orthogonal to $r_{i}$, Equation (4) is approximated by

(10) $$Z_{i}^{\left(s\right)}\leftarrow \frac{x_{i}^{T}R^{T}r_{i}r_{i}^{T}\sum_{j\in N_{i}}p_{i,j}\left(X_{j}^{\left(r\right)}-T\right)}{x_{i}^{T}R^{T}r_{i}r_{i}^{T}Rx_{i}}=\frac{x_{i}^{T}R^{T}\sum_{j\in N_{i}}p_{i,j}\left(X_{j}^{\left(r\right)}-T\right)}{∥Rx_{i}{∥}^{2}},$$

which is equivalent to Equation (6) based on the point-to-point distance.

On the other hand, if $n_{j}$ is nearly parallel with $r_{i}$, Equation (4) is approximated by

(11) $$Z_{i}^{\left(s\right)}\leftarrow \frac{x_{i}^{T}R^{T}\sum_{j\in N_{i}}p_{i,j}n_{j}n_{j}^{T}\left(X_{j}^{\left(r\right)}-T\right)}{x_{i}^{T}R^{T}\sum_{j\in N_{i}}p_{i,j}n_{j}n_{j}^{T}Rx_{i}},$$

which is purely based on the point-to-plane distance.

If $X_{i}^{\left(s\right)}$ has no point satisfying $∥d_{i,j}∥<\tau$, Equation (4) is not constructed for such $X_{i}^{\left(s\right)}$ without valid closest points. To attract such points to those refined by valid closest points, Choi et al. [26] use a regularization method that moves the source points as rigidly as possible toward the reference points. As the cost function has been designed to preserve the original structure of $S_{s}$, the depth measurement error in $S_{s}$ is hardly reduced by the method if the overlap between $S_{s}$ and $S_{r}$ is small.

To treat every source point uniformly, we can regard $S_{r}\cup S_{s}^{\prime }$ as the reference point cloud instead of $S_{r}$, where $S_{s}^{\prime }$ denotes the duplicate of $S_{s}$. Assuming that the distance between neighboring points in $S_{s}$ is shorter than τ, $N_{i}$ is not an empty set for all *i*. In this case, however, most of the *K*-closest points will tend to be selected from $S_{s}^{\prime }$. Such closest points hardly contribute to reducing the distance between $S_{r}$ and $S_{s}$. To avoid this problem, we select two sets of *K*-closest points from $S_{s}^{\prime }$ and $S_{r}$ independently.

With the two sets of closest points, our cost function is defined as

(12) $$E_{i}=\sum_{j\in N_{i}^{\left(r\right)}}p_{i,j}d_{i,j}^{T}M_{i,j}d_{i,j}+\alpha \sum_{k\in N_{i}^{\left(s\right)}}p_{i,k}d_{i,k}^{T}M_{i,k}d_{i,k},$$

where $N_{i}^{\left(r\right)}$ and $N_{i}^{\left(s\right)}$ are the index sets of the *K*-closest points to ${\hat{X}}_{i}^{\left(s\right)}$ and $X_{i}^{\left(s\right)}$ in $S_{r}$ and $S_{s}^{\prime }$, respectively. We note that $d_{i,k}=X_{k}^{\left(s\right)}-X_{i}^{\left(s\right)}$ as the transformation from $S_{s}$ to its duplicate $S_{s}^{\prime }$ is the identity transformation.

A positive constant α controls the effect of the *K*-closest points from $S_{s}^{\prime }$. As we want their effect to be small if $N_{i}^{\left(r\right)}$ is not an empty set, a reasonable choice of α is a small positive number, such as 0.01. We investigate the effect of α by varying its value from 0.01 to 1 in Section 4.

Assuming that all points in $S_{r}\cup S_{s}^{\prime }$ are fixed, we can derive the closed-form solution that minimizes Equation (12). Equation (13) is the consequent update equation with the two sets of *K*-closest points.

(13) $$Z_{i}^{\left(s\right)}\leftarrow \frac{x_{i}^{T}\left(R^{T}\sum_{j\in N_{i}^{\left(r\right)}}p_{i,j}M_{i,j}\left(X_{j}^{\left(r\right)}-T\right)+\alpha \sum_{k\in N_{i}^{\left(s\right)}}p_{i,k}M_{i,k}X_{k}^{\left(s\right)}\right)}{x_{i}^{T}\left(R^{T}\sum_{j\in N_{i}^{\left(r\right)}}p_{i,j}M_{i,j}R+\alpha \sum_{k\in N_{i}^{\left(s\right)}}p_{i,k}M_{i,k}\right)x_{i}}.$$

The proposed method can be extended to a set of L+1 point clouds by iteratively registering a point cloud to the union of the point clouds. Choi et al. [26] proposed an algorithm for the extension, and Algorithm 1 shows the algorithm with a slight modification to use Equation (13). In Algorithm 1, ${\hat{S}}_{i}$ is the transformed $S_{i}$ to the reference frame using the pose parameters $R_{i}$ and $T_{i}$. ${ITER}_{max}$ is the number of cycles of depth-filtering operations. We set ${ITER}_{max}$ to two throughout this paper, as in [26]. This setting allows every point, except for those in $S_{0}$, to be filtered twice. The points in $S_{0}$ are filtered once under this setting.
**Algorithm 1:** Multiview depth refinement algorithm.
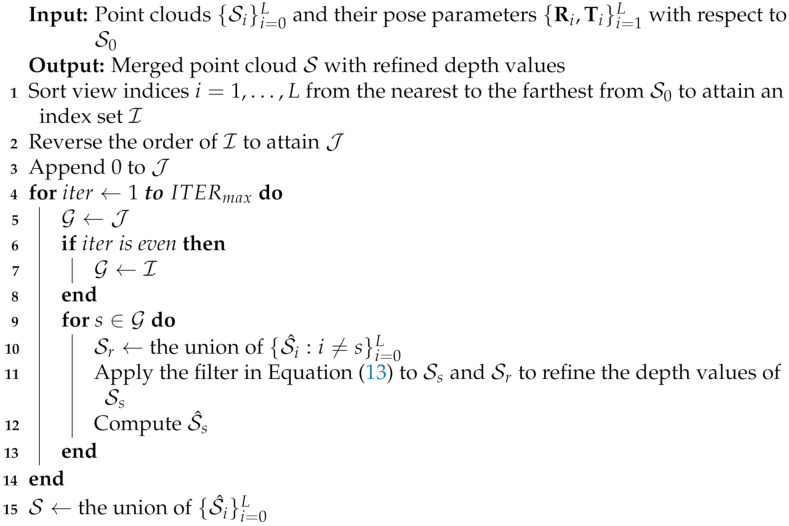


## 4. Results

This section provides experimental results. For a comparison to previous work, we use the synthetic and real-world datasets of Choi et al. [26]. We provide quantitative results using the synthetic dataset and qualitative results using the real-world dataset.

**Figure 2:**
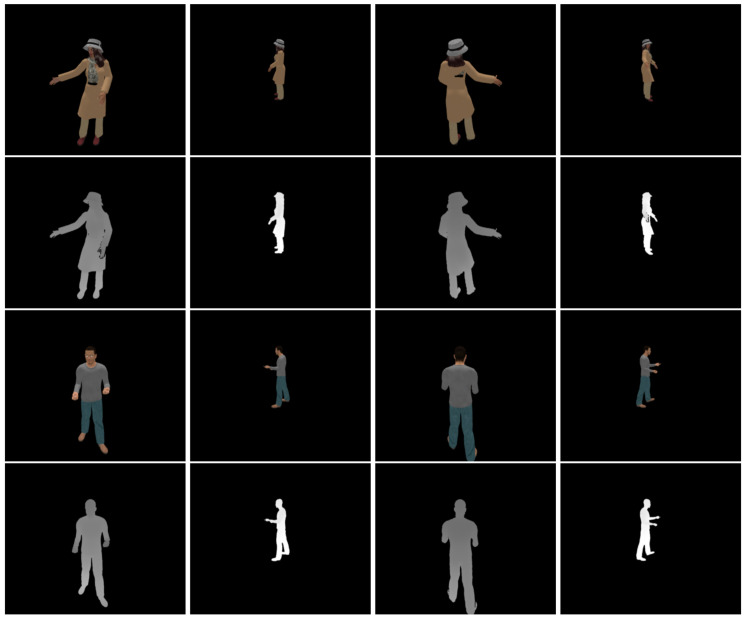
Sample RGB-D images in the synthetic dataset of Choi et al. [26]. (**First row**): Color images of the female model. (**Second row**): Depth images of the female model. (**Third row**): Color images of the male model. (**Fourth row**): Depth images of the male model. (**First column**): View 0. (**Second column**): View 3. (**Third column**): view 6. (**Fourth column**): View 9. The intensity of the depth images is linear with depth values.

The synthetic multiview RGB-D dataset [26] was constructed by rendering graphics models of the pose-varying human model dataset [38]. Twenty mesh models of different poses were sampled from a male (models 0–199) and a female (models 6800–6999) appearance, respectively. The number of views is twelve (L=11), and the distance to the models ranges from 1.5 m to 3 m. The 0th and 6th views are the closest, and the 3rd and 9th views are the farthest. The standard deviation of depth noise is approximately in proportion to the squared depth values [29], and such realistic noise was added to the rendered depth images. The standard deviation of the noise ranges from 0.5 cm to 2.2 cm. The 3rd and 9th depth images suffer from the highest noise level, while the 0th and 6th depth images suffer from the lowest noise level. The ground-truth camera pose parameters are provided with the dataset. Thus, we can compare the registered output depth images to the registered ground-truth depth images with no pose error. Figure 2 shows sample RGB-D images from the synthetic dataset.

We compare the proposed method to three existing methods [26,28,39] and two extreme variants of the proposed method. We implemented the bilateral filter for point clouds [28], which is referred to as the Bilateral filter. We applied the guided image filter [39] to point cloud filtering, which is referred to as the Guided filter. The guided image filter has shown high performance not only in image filtering but also in cost volume filtering for stereo matching [40]. In our implementation, the parameters of the two filters were set similarly to ours. For example, the maximum number of neighbors was set to 10 with the same threshold τ = 4 cm. If the number of neighbors was less than 5, then at least five neighbors were used. As the filters were applied to the union of all multiview point clouds, this setting gave the filters approximately the same number of neighbors as the proposed method, which found a maximum of five closest points from the source and reference point clouds, respectively. The filters were applied twice so that each point would be filtered twice as in our method. On the other hand, we used the results of Choi et al. reported in [26] without re-implementation.

Our proposed Algorithm 1 is referred to as Filter adaptive. Filter p2p is a variant of Filter adaptive, where only point-to-point distances are used. Filter p2l is another variant, where only point-to-plane distances are used. The two variants are obtained by fixing $c_{i,j}$ in Equation (8) to either 0 or 1. With the results of these variants, we can understand the effect of the proposed adaptive cost function.

### 4.1. Results on the Synthetic Dataset

The synthetic data set provides perturbed pose parameters, where five different rotational and translational perturbations were applied to the ground-truth rotation matrices and translation vectors with rotation angles of $2^{\circ }$ to $10^{\circ }$ and translation lengths of 5 cm to 25 cm, respectively. Regarding the perturbed pose parameters as the outputs of the inaccurate calibration or global registration, the IKCP algorithm for pose refinement [26] was applied to reduce the registration error. To simulate a practical use case of the proposed method, the output pose parameters of the local pose refinement algorithm and the noisy depth images were used as input in this section, unless otherwise mentioned. The registration method of merging noisy point clouds with the estimated pose parameters is referred to as initial.

The accuracy was measured by computing the RMSE between a filtered source point cloud and its corresponding ground-truth source point cloud:

(14) $$\mathrm{RMSE}=\sqrt{\frac{1}{N_{s}}\sum_{i=1}^{N_{s}}∥R_{s,gt}X_{i,gt}^{\left(s\right)}+T_{s,gt}-R_{s}X_{i}^{\left(s\right)}-T_{s}{∥}^{2}},$$

where *s*, ranging from 0 to *L*, is the index of the source point cloud. $R_{s,gt}$ and $T_{s,gt}$ are the ground-truth pose parameters of the *s*th view, while $R_{s}$ and $T_{s}$ are the estimated pose parameters by the local pose refinement algorithm. $X_{i,gt}^{\left(s\right)}$ is the *i*th 3D point from the *s*th ground-truth depth image, while $X_{i}^{\left(s\right)}$ is its corresponding filtered 3D point.

**Figure 3:**
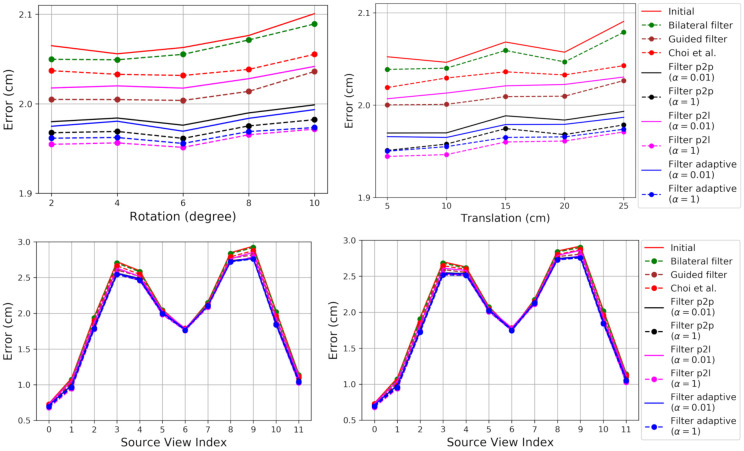
Evaluation of the depth refinement algorithms on the synthetic dataset of Choi et al. [26]. The algorithms are initialized with the estimated transformations by a local pose refinement algorithm [26] applied to the transformations that are perturbed away from the true pose. (**Top**): Error according to different perturbation levels in the rotational (**left**) and translational (**right**) components. (**Bottom**): Error according to the source view index with perturbation levels $10^{\circ }$ (**left**) and 25 cm (**right**). The plot shows the median RMSE. Lower is better. Best viewed in color.

Figure 3 shows the RMSE. The proposed method and its variants consistently result in lower errors than the existing methods [26,28,39], except for Filter p2l with α=0.01. One of the differences of the proposed Filters from the method of Choi et al. is the closest points within source point clouds, which are used for intra-point-cloud filtering. The reduced noise by the intra-point-cloud filtering is one of the contributions to the reduced RMSE. Figure 3 shows that the proposed method is more effective for the views with more noise, showing larger performance gaps from Initial.

The RMSE with α=1 is consistently lower than with α=0.01. A large α denotes more intra-point-cloud filtering and relatively less inter-point-cloud filtering. The intra-point-cloud filtering is not affected by the error in the estimated pose. Thus, a large α can provide better results in the presence of a pose error.

**Figure 4:**
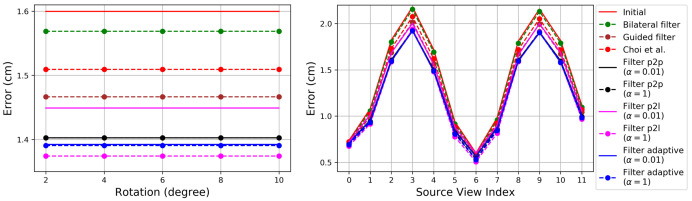
Evaluation of the depth refinement algorithms on the synthetic dataset of Choi et al. [26]. The algorithms are initialized with the ground-truth pose parameters, so there is no pose estimation error. (**Left**): Error according to different perturbation levels in the rotational component. The errors are constant without pose estimation error. The main cause of the registration errors are depth errors. (**Right**): Error according to the source view index.

If the multiview system has been calibrated accurately, one can expect low pose error. To compare the performances in the absence of pose errors, we applied the methods to the point clouds in their ground-truth poses. Figure 4 shows the results. Filter p2p and Filter adaptive show consistent results, irrespective of the choice of α. It is interesting to notice that Filter p2l with α=0.01 provides better results than Guided filter. We conjecture that this is due to the fact that the inter-point-cloud closest points are now more accurate neighbors for filtering. However, Filter p2l still shows worse results with α=0.01 than with α=1.

Filter p2l suffers from the instability problem addressed in this paper. A source point and its intra-point-cloud closest points tend to have similar ray directions and surface-normal directions. An RGB-D camera cannot measure the depth of a surface whose normal direction is orthogonal to its ray direction, so the normal directions are difficult to make orthogonal to the ray directions as long as the depth measurements exist. Thus, the stability of Equation (13) for Filter p2l increases with α, reducing the RMSE.

**Figure 5:**
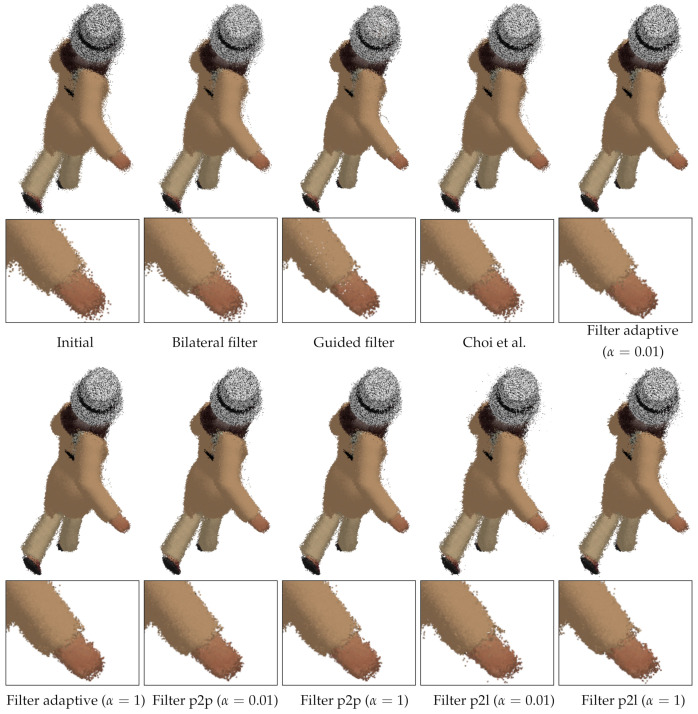
Point cloud rendering results. (**First and third rows**): Merged point clouds. (**Second and fourth rows**): Magnified hand regions. We note that neither a preprocessing nor a postprocessing method has been applied to the results.

Figure 5 shows merged point clouds obtained by different depth refinement methods. The results were obtained from the inputs with 25 cm perturbation levels. The qualitative results are consistent with the quantitative results in Figure 3. Filters with α=1 show the best results with greatly reduced noise. Filter p2l with α=0.01 shows the worst result among Filters.

The running time of the proposed Algorithm 1 is reported in Table 1. The running time was measured on a computer running Ubuntu 18.01 with an AMD Ryzen Threadripper 1920X 12-core processor and 128 GB of RAM. In Table 1, all the algorithms are based on our unoptimized Python implementation. Therefore, the running times are appropriate only for relative comparison. Among the Filters, Filter p2p is the most efficient and Filter adaptive is the most demanding. As Filter adaptive computes two different kinds of projection matrices, it requires more computation time. The intra-point-cloud closest point search can be conducted only once, assuming that they do not change in the whole process. This assumption can reduce the computation time. However, our current implementation does not rely on the assumption. The running times of Bilateral filter and Guided filter are approximately half of that of Filter p2p. This is mainly due to the fact that the proposed method conducts the *K*D tree search once more for each filtering.

### 4.2. Results on the Real-World Dataset

**Figure 6:**
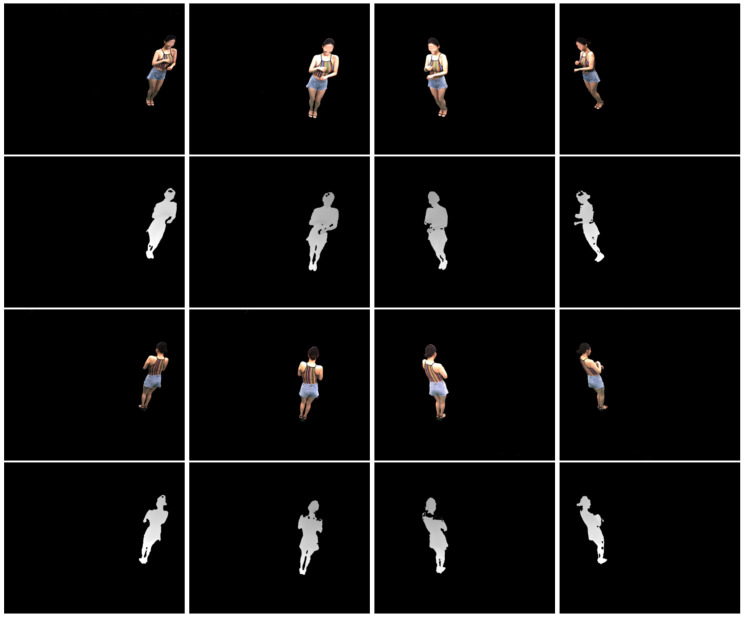
Real multiview RGB-D images [26]. (**First and third rows**): Color images of the model. (**Second and fourth rows**): Depth images of the model. The intensity of the depth images is linear with depth values. The face regions in the front views have been blurred to protect the model’s identity.

In this section, we describe the application of the proposed method to the real-world dataset [26]. The dataset is composed of eight RGB-D images, as shown in Figure 6. The dataset was captured under accurate calibration, and the extrinsic parameters were further refined by the local pose refinement method [26]. Thus, we can expect that the error in the estimated pose will be less than that of the synthetic dataset. Since the dataset was not captured with accurate laser scanners, an exact quantitative evaluation is not available.

**Figure 7:**
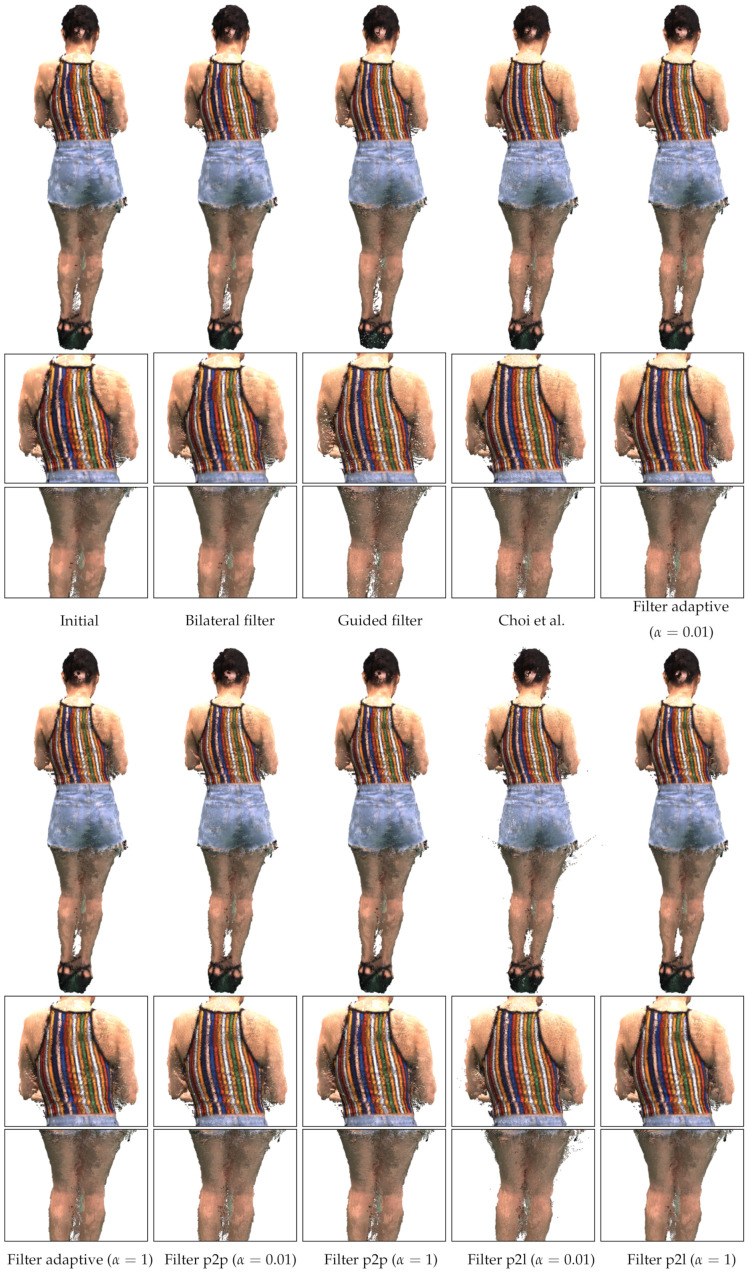
Point cloud rendering results. (**First and fourth rows**): Merged point clouds. (**Second, third, fifth and sixth rows**): Magnified regions. We note that neither a preprocessing nor a postprocessing method has been applied to the results.

Figure 7 shows merged point clouds obtained using different depth refinement methods. The best method for the results is subjective. If we focus on the stripe patterns on the back, Filter p2l with α=0.01 and Choi et al. [26] show the best results. Filters with α=1 do not improve the stripe pattern of Initial as much as those with α=0.01. The visual quality of a merged point cloud highly relies on the distance between similarly colored points across point clouds. The small α increases the effect of the inter-point-cloud filtering, so the inter-point-cloud distance is reduced. In addition, with the accurate pose parameters, α=0.01 provides quantitatively equivalent results to α=1, except for Filter p2l, as shown in Figure 4.

In contrast, if we focus on the artifacts near the outer thighs, Filter p2l with α=0.01 shows the worst result. The errors caused by the numerical instability are reduced by increasing α, as discussed in Section 4.1. However, neither the postprocessing method of Choi et al. [26] nor the intra-point-cloud filtering of Filter p2l completely removes the errors. In contrast, Filter adaptive suffers less from the outer thigh errors than Filter p2l, showing the effectiveness of the adaptive combination of the projected distances.

## 5. Conclusions and Future Work

We proposed a unified depth-filtering method for colored point-cloud registration. Within the IKCP framework for depth refinement, our cost function is constructed by adaptively combining two different projected distances to prevent the numerical instability of using the point-to-plane distance only. We extended the closest point search range to include the source point cloud. This extension reduced the registration error further by reducing the depth errors. It also improved the numerical stability of using the point-to-plane distance.

Finding the balance between the intra- and the inter-point-cloud filtering is the key for improving the registration accuracy and the visual quality of the merged point cloud. In our future research, we will investigate an adaptive method for finding the balance.

## Figures and Tables

**Table 1:** Average running time (seconds).

Bilateral filter	220.02
Guided filter	201.17
Choi et al.	596.80
Filter adaptive	857.78
Filter p2p	432.26
Filter p2l	684.93

## Data Availability

Data sharing not applicable.

## Abbreviations

The following abbreviations are used in this manuscript:

ICP	Iterative Closest Point
IKCP	Iterative *K* Closest Point
RGB-D	Red Green Blue-Depth
RMSE	Root Mean Square Error
